# Supplementary material for: Enhancing PM2.5 prediction by mitigating annual data drift using wrapped loss and neural networks
Source: PLoS One. 2025 Feb 11;20(2):e0314327. doi: 10.1371/journal.pone.0314327 (PMC11813127; doi:10.1371/journal.pone.0314327)
Supplement: S1 Table — (PDF) [file pone.0314327.s001.pdf]

Table 1: The base model and Wrap loss model performance for RMSE ($\mu g/m^3$) value

Station name(3): Guting									
Model	1h	8h	16h	24h	32h	40h	48h	56h	64h
$BILSTM$	3.38	8.49	9.28	9.34	9.55	9.63	9.77	9.85	9.931
$WBILSTM$	4.51	9.26	9.26	9.34	9.49	9.58	9.73	9.87	9.93
$LSTM$	3.35	8.42	9.279	9.23	9.79	9.708	9.74	9.90	9.93
$WLSTM$	4.51	8.41	9.52	9.36	9.89	9.86	9.75	9.82	9.931
$LS-AT$	4.23	6.89	7.3	7.79	8.29	8.43	8.81	8.96	9.72
$WLS-AT$	4.07	6.38	6.85	7.26	7.11	7.84	8.01	8.07	8.87
CNN	4.39	9.43	9.87	9.90	10.27	10.75	11.07	12.39	12.71
$WCNN$	4.01	6.89	7.42	7.51	7.71	8.23	8.42	8.15	8.2
FLC	4.19	5.94	6.44	6.88	6.97	7.62	8.29	8.51	8.67
$WFLC$	4.12	5.73	6.42	6.72	7.57	7.61	8.16	8.07	7.94
BLC	4.04	5.87	6.67	6.67	6.94	7.39	7.37	8.15	8.86
$WBLC$	5.84	6.57	6.88	7.11	7.27	7.46	7.86	7.97	8.73
Station name(4): Linkou									
Model	1h	8h	16h	24h	32h	40h	48h	56h	64h
$BILSTM$	3.87	8.28	9.51	10.05	10.17	10.21	10.29	10.34	10.43
$WBILSTM$	3.18	8.14	9.18	9.52	9.65	10.12	10.09	10.15	10.16
$LSTM$	5.05	9.06	9.47	10.03	10.2	10.25	10.31	10.37	10.42
$WLSTM$	3.77	9.04	9.59	9.81	10.02	10.11	10.18	10.25	10.28
$LSTM-AT$	3.44	7.74	8.79	9.29	9.83	10.16	10.42	10.25	10.48
$WLS-AT$	3.08	7.45	8.864	9.42	9.45	10.15	10.26	10.23	10.28
CNN	3.04	7.72	9.00	9.70	10.24	10.36	10.48	10.43	11.02
$WCNN$	3.08	7.7	8.89	9.64	10.14	10.23	10.39	10.222	11.05
FLC	3.16	6.32	7.37	8.22	8.58	8.9	8.82	9.93	9.93
$WFLC$	3.16	6.32	7.37	8.22	8.58	8.9	8.82	9.93	9.93
BLC	3.31	6.34	7.36	7.95	8.42	8.81	9.03	9.28	9.51
$WBLC$	3.25	6.23	7.39	8.08	8.63	8.8	8.94	9.21	9.28
Station name(5): Sanchong									
Model	1h	8h	16h	24h	32h	40h	48h	56h	64h
$BILSTM$	6.84	9.12	9.52	9.7	9.82	9.89	9.91	9.93	9.99
$WBILSTM$	7.17	8.98	9.47	9.6	9.55	9.4	9.74	9.79	9.87
$LSTM$	6.97	9	9.64	9.69	9.78	9.87	9.91	9.98	10.21
$WLSTM$	7.01	9.53	9.38	9.48	9.59	9.64	9.92	9.97	10.01
$LS-AT$	4.44	10.11	9.98	9.18	9.24	10.09	10.15	10.23	10.85
$WLS-AT$	4.12	8.79	8.96	9.1	9.24	9.73	9.67	9.73	9.73
CNN	4.10	8.85	9.02	9.09	9.28	9.71	9.63	10.22	10.39
$WCNN$	4.122	7.32	8.69	9.11	9.28	9.05	9.57	10.21	10.35
FLC	4.18	8.52	9.02	9.05	9.66	9.94	9.98	10.04	10.38
$WFLC$	4.33	8.87	9.17	9.31	9.49	9.44	9.57	9.65	9.67
BLC	4.09	8.15	8.47	8.89	9.56	9.76	9.72	9.85	9.99
$WBLC$	4.1	8	8.57	9.35	9.54	9.61	9.07	9.62	9.9

Table 2: The base model and propose model performance for MAE($\mu g/m^3$) value

Station name(1): Banqiao									
Model	1h	8h	16h	24h	32h	40h	48h	56h	64h
$BILSTM$	2.46	5.64	6.76	7.22	7.65	7.86	7.87	7.86	8.2
$WBILSTM$	2.45	5.59	6.55	7.24	7.75	7.81	7.92	7.84	7.99
$LSTM$	2.47	5.6	6.79	7.17	7.95	7.87	7.87	7.97	7.92
$WLSTM$	2.44	5.45	6.35	7.05	7.43	7.69	7.73	7.94	7.82
$LSTM-AT$	2.46	5.64	6.76	7.22	7.65	7.86	7.87	7.86	8.2
$WLSTM-AT$	2.44	5.45	6.35	7.05	7.43	7.69	7.73	7.94	7.82
CNN	2.46	6.13	6.77	7.12	7.26	7.86	7.9	7.86	7.93
$WCNN$	2.45	5.73	6.83	7.13	7.18	7.84	7.86	7.9	7.91
FLC	2.47	4.57	5.49	6.09	6.62	6.78	7.18	7.39	7.49
$WFLC$	2.47	4.5	5.45	6.1	6.57	6.61	7.01	6.95	7.29
BLC	2.47	4.5	5.51	6.04	6.12	6.6	7.07	7.48	7.56
$WBLC$	2.47	4.58	5.43	6.09	6.01	6.55	6.92	7.12	7.3
Station name(2): Cailiao									
$BILSTM$	2.14	5.22	6.2	6.54	7.42	7.52	7.24	7.57	7.66
$WBILSTM$	2.14	5.23	5.75	6.68	6.95	7.43	7.29	7.56	7.66
$LSTM$	2.15	5.2	6.14	6.66	7.04	7.23	7.49	7.44	7.55
$WLSTM$	2.13	5.18	6.18	6.61	7.2	7.27	7.41	7.41	7.42
$LSTM-AT$	2.14	5.22	6.2	6.54	7.42	7.52	7.85	7.92	7.99
$WLSTMAT$	2.45	5.32	6.38	6.97	6.95	7.42	7.44	7.57	7.56
CNN	2.13	5.33	6.19	6.6	6.56	7.1	7.23	7.48	7.54
$WCNN$	2.13	5.31	6.07	6.61	6.59	7.4	7.06	7.27	7.36
FLC	2.15	4.13	5.02	5.47	6.03	6.43	6.47	6.75	6.86
$WFLC$	2.14	4.09	4.96	5.54	5.8	5.83	6.09	6.46	6.83
BLC	2.16	4.1	5.03	5.46	5.6	6.23	6.49	6.7	6.73
$WBLC$	2.14	4.11	5.01	5.45	5.44	5.89	6.3	6.47	6.62
Station name(3): Songshan									
$BILSTM$	3.46	6.13	6.71	6.91	7.48	7.7	7.57	7.87	7.82
$WBILSTM$	3.41	6.03	7.03	7.11	7.44	7.67	7.48	7.85	7.72
$LSTM$	3.43	6.03	6.7	6.9	7.27	7.58	7.43	7.94	7.72
$WLSTM$	3.42	6.04	6.73	6.97	7.45	7.55	7.57	7.86	7.88
$LSTM-AT$	3.46	6.13	6.71	6.91	7.48	7.7	7.57	7.87	7.88
$WLSTM-AT$	3.46	5.33	6.54	6.7	6.67	7.22	7.43	7.64	7.55
CNN	3.44	6.47	7.01	6.9	6.96	7.58	7.64	7.95	7.95
$WCNN$	3.46	6.35	6.84	6.69	6.85	7.57	7.47	7.37	7.76
FLC	3.44	5.29	5.87	6.48	6.76	7.03	7.16	7.27	7.23
$WFLC$	3.44	5.23	6.05	6.42	6.73	6.74	6.74	7.07	7.13
BLC	3.47	5.25	5.92	6.4	6.39	6.93	6.93	7.19	7.38
$WBLC$	3.45	5.22	5.94	6.38	6.32	6.73	6.94	7.14	7.29

Table 3: The base model and propose model for MAPE value(%)

Station name(8): Cailiao									
$BILSTM$	18.17	36.06	41.19	44.6	43.18	43.8	45.75	45.33	45.99
$WBILSTM$	19.26	36.97	42.01	42.46	42.41	44.54	44.74	45.17	44.74
$LSTM$	17.76	29.41	35.21	38.1	41.29	42.23	42.08	44.43	42.82
$WLSTM$	17.63	29.62	33.81	37.26	38.94	41.97	41.56	41.35	42.26
$LSTM-AT$	17.65	28.54	34.06	38.02	36.81	40.3	41.24	41.43	42.16
$WLSTM-AT$	17.42	28.45	33.04	39.02	38.81	39.69	41.22	42.14	41.51
CNN	18.17	36.06	41.19	44.6	43.18	43.8	45.75	45.33	45.99
$WCNN$	19.26	36.97	42.01	42.46	42.41	44.54	44.74	45.17	44.74
FLC	17.76	29.41	35.21	38.1	41.29	42.23	42.08	44.43	42.82
$WFLC$	17.63	29.62	33.81	37.26	38.94	41.97	43.56	41.35	42.26
BLC	17.65	28.54	34.06	38.02	36.81	40.3	41.24	41.43	42.16
$WBLC$	17.42	28.45	33.04	39.02	38.81	39.69	41.22	42.14	41.51
Station name(8): Guting									
Model	1h	8h	16h	24h	32h	40h	48h	56h	64h
$BILSTM$	26.4	34.4	35.46	36.12	38.15	39.61	42.3	42.25	46.19
$WBILSTM$	27.48	34.2	33.45	34.87	38.77	38.7	42.58	41.35	45.55
$LSTM$	26.31	38.48	37.68	41.41	41.28	42.29	44.6	43.05	45.75
$WLSTM$	26.14	39.33	38.28	42.87	41.33	41.12	42.24	42.17	43.7
$LS-AT$	26.4	42.4	42.46	41.12	42.15	42.61	42.3	42.25	44.19
$WLS-AT$	25.87	38.89	42.73	41.19	42.96	43	41.78	42.52	42.39
CNN	28.18	39.77	43.24	40.64	41.19	42.55	43.2	43.58	45.73
$WCNN$	27.24	39.76	42.93	41.22	40.79	45.01	42.96	43.22	43.86
FLC	26.71	36.57	37.12	38.2	38.85	39.46	41.33	41.68	41.61
$WFLC$	25.98	34.03	38.14	38.64	38.21	38.14	40.93	41.08	41.06
BLC	26.71	36.57	37.12	38.2	37.85	39.46	41.33	41.68	41.11
$WBLC$	25.31	35.62	37.2	37.35	37.29	39.89	39.66	40.11	40.11
Station name(8): Xizhi									
Model	1h	8h	16h	24h	32h	40h	48h	56h	64h
$BILSTM$	23.95	41.57	44.75	46.18	46.26	48.09	49.68	50.24	51.44
$WBILSTM$	23.79	39.4	44.36	46.86	46.68	48.89	49.29	49.89	50.54
$LSTM$	24.04	41.09	45.33	46.94	48.56	49.8	50.18	49.36	50.29
$WLSTM$	23.76	41.37	46.91	46.88	49.02	50.14	53.23	49.3	49.88
$LSTM-AT$	23.95	41.57	47.75	46.18	49.26	49.09	49.68	50.24	51.44
$WLS-AT$	23.78	43.49	46.6	46.43	48.77	49.47	49.52	49.11	50.35
CNN	23.41	42.17	47.37	47.45	47.3	49.12	50.41	49.89	50.8
$WCNN$	23.34	42.59	47.16	46.78	47.68	49.74	50.11	49.89	50.79
FLC	24.36	37.14	41.68	43.77	46.69	47.35	48.39	48.13	48.84
$WFLC$	23.96	36.45	41.71	45.46	44.62	47	45.93	48.68	47.99
BLC	24.01	38.05	41.53	44.05	42.99	47.1	47.38	49.09	48.59
$WBLC$	23.91	37.4	42.23	43.48	43.64	47.7	47.32	47.91	47.21

Table 4: The Average for the base model and propose model performance for MAPE value(%)

Station name(8): Cailiao									
$BILSTM$	26.07	42.09	43.32	44.32	45.85	46.68	47.24	47.26	48.11
$WBILSTM$	25.42	41.01	44.61	44.8	46.11	46.42	46.56	46.67	46.85
$LSTM$	26.32	41.29	44.14	44.68	46.05	46.48	47.1	47.17	48.41
$WLSTM$	25.43	40.99	43.62	44.92	46.34	46.37	46.84	47.05	47.34
$LSTM-AT$	26.05	41.74	43.16	44.58	45.26	46.81	47.51	47.78	49.12
$WLSTM-AT$	24.93	38.76	42.88	44.44	44.66	46.83	47.27	47.41	48.28
CNN	18.17	36.06	41.19	44.6	43.18	43.8	45.75	45.33	45.99
$WCNN$	19.26	36.97	42.01	42.46	42.41	44.54	44.74	45.17	44.74
FLC	17.76	29.41	35.21	38.1	41.29	42.23	42.08	44.43	42.82
$WFLC$	17.63	29.62	33.81	37.26	38.94	41.97	43.56	41.35	42.26
BLC	17.65	28.54	34.06	38.02	36.81	40.3	41.24	41.43	42.16
$WBLC$	17.42	28.45	33.04	39.02	38.81	39.69	41.22	42.14	41.51
