# Supplementary material for: Enhancing PM2.5 prediction by mitigating annual data drift using wrapped loss and neural networks
Source: PLoS One. 2025 Feb 11;20(2):e0314327. doi: 10.1371/journal.pone.0314327 (PMC11813127; doi:10.1371/journal.pone.0314327)
Supplement: Attachment — Submitted filename: Response to Reviewers.pdf [file pone.0314327.s002.pdf]

[Responses Academic Editors and Reviewers]

Point-by-Point Responses and the Reviewer Comments of

On the Data Shifting: Predicting PM2.5 levels by using CNN based neural network

Md Khalid Hossen*, Yan-Tsung Peng, Meng Chang Chen
Plos Reviewers Response

---

1. Academic Editors

Comment **1: (general comment) Please ensure that your manuscript meets PLOS ONE's style requirements, including those for file naming. The PLOS ONE style templates can be found at `https://journals.plos.org/plosone/s/file?id=wjVg/PLOSOne_formatting_sample_main_body.pdf` and `https://journals.plos.org/plosone/s/file?id=ba62/PLOSOne_formatting_sample_title_authors_affiliations.pdf`**

Response I have followed the the above format in my manuscript

Comment **) 2:Please note that PLOS ONE has specific guidelines on code sharing for submissions in which author-generated code underpins the findings in the manuscript. In these cases, all author-generated code must be made available without restrictions upon publication of the work. Please review our guidelines at `https://journals.plos.org/plosone/s/materials-and-software-sharingloc-sharing-code` and ensure that your code is shared in a way that follows best practice and facilitates reproducibility and reuse.**

Response Definitely, I will share the source code and so that it can reproduce.

Comment **3) Thank you for stating the following financial disclosure:**

"This document is the result of the research project funded by the National Science and Technology Council of Taiwan, under the grant NSC 109-2119-M-001-010-A." Please state what role the funders took in the study. If the funders had no role, please state: "The funders had no role in study design, data collection and analysis, decision to publish, or preparation of the manuscript." If this statement is not correct you must amend it as needed. Please include this amended Role of Funder statement in your cover letter; we will change the online submission form on your behalf.

Response This study was supported by National Science and Technology Council of Taiwan, under the grant NSC 109-2119-M-001-010-A. No additional external funding was received for this study. The funders had no role in study design, data collection and analysis, decision to publish, or preparation of the manuscript.

Comment **4) Specific Comment 4: Please note that funding information should not appear in the Acknowledgments section or other areas of your manuscript. We will only publish funding information present in the Funding Statement section of the online submission form. Please remove any funding-related text from the manuscript.**

Response **I already removed from the Acknowledge sections.**

I already removed from the Acknowledge sections.

Comment **5) Specific Comment 5: When completing the data availability statement of the submission form, you indicated that you would make your data available on acceptance. We strongly recommend all authors decide on a data sharing plan before acceptance, as the process can be lengthy and hold up publication timelines. Please note that, though access restrictions are acceptable now, your entire data will need to be made freely accessible if your manuscript is accepted for publication. This policy applies to all data except where public deposition would breach compliance with the protocol approved by your research ethics board. If you are unable to adhere to our open data policy, please kindly revise your statement to explain your reasoning and we will seek the editor's input on an exemption. Please be assured that, once you have provided your new statement, the assessment of your exemption will not hold up the peer review process.**

Response The data we collected are called EPA data and shared in the below link `https://1drv.ms/f/c/615854b8e839ca3e/EpO3bYopjRBHjEWAWlO0WtIBU0b2NPiGliOhrsdKMxRCwA?e=RfX5z7` and after preprocessing the EPA data collected only for Taipei area and the evaluation process used this datasets `https://1drv.ms/f/c/615854b8e839ca3e/EvywgMKXuJRLian31QPWT0IBVSCWtKVWc9fu4vrqaNEKCQ?e=txnbOZ`

Comment **6. Please amend either the abstract on the online submission form (via Edit Submission) or the abstract in the manuscript so that they are identical.**

Response I have edited the abstract from the online submission.

Comment **7. Please ensure that you refer to Figure 12 in your text as, if accepted, production will need this reference to link the reader to the figure.**

Response I have confirmed about figure 12 and it's included in the manuscript.

Comment **8. We note you have included a table to which you do not refer in the text of your manuscript. Please ensure that you refer to Tables 4 and 8 in your text; if accepted, production will need this reference to link the reader to the Table.**

Response I have confirmed Tables 4 and 8 so that their text is included in the manuscript.

2. Reviewer Responses

3. Reviewer 1

Comment **Reviewer 1: (general comment) This omission raises questions about the thoroughness with which the proposed models are presented and their potential advantages over existing methods. It is not clear if the integration of advanced neural network architectures and novel loss functions for improved accuracy in PM2.5 prediction is also a paper's contribution as the title states. Clarify.**

Response I have discussed in detail about contributions and clarified the contributions in the end of the Abstract Section.

> In this work, the proposed model and the loss function are both contributions for this work to yanking the data drifting scenario. The CNN-Attention-based model solves the data drifting issue, and the wrap loss function also helps alleviate the data drifting problem with model training and works for the baseline model and neural network models to achieve more correct results.

Comment **2) Specific Comment 2: However, the absence of a discussion on LSTM networks with attention mechanisms raises questions about the comprehensive coverage of existing methodologies and their comparative analysis within the study.**

Response After getting feedback from the reviewers, it is added the LSTM attention-based model and included in the Model Designs section. It found that their result could not compete with our proposed model. The following sentence is added to the Results and Discussion section. We have discussed LSTM attention with their loss functions too.

> In the LSTM-attention model is also applied to the time series [43] prediction, in our work we have applied for hourly PM2.5 prediction its results are included and compared with other models.

Comment **3) Specific Comment 3: Furthermore, the paper references work number 33 in the state of the art section on transfer learning, which also proposes CNNs with attention mechanisms for forecasting tasks. This reference necessitates a clear delineation of how the current paper's approach diverges from the methodologies previously outlined. The distinction appears to lie solely in the specific focus on data-shifting strategies and the implementation of a novel loss function. Please Clarify in the text.**

Response Line 33, I have added our work necessities and compared them with the work that has been used as a reference outlined in [33].

> ~~They trained their model using data from the last four years and used it to predict the PM2.5 level of Taipei City for the next year without addressing the data drifting issue. Taking advantage of abundant data to tackle data drifting challenges through transfer learning, they introduced a CNN attention-based model designed to make hourly predictions from time series data, 144 thereby improving the prediction performance and overcoming domain adaptation 145 challenges specific to time series data.~~This work focused on solving the problem where the new city developed and did not have enough data, and in some cases, they did not have enough pollutant observation of pollutant sources in the area. The authors transferred the knowledge from one enriched city Beijing to another city Baoding. They needed the source city and a target city for transfer learning, but in our proposed model, it does not require any source or target city. Also, they did not discuss the data drifting issues. Our proposed model has one more advantage that it predicts from EPA data and weather data from the last couple of years and also includes domain adaptation

Comment **4) Specific Comment 4: In terms of the presentation of results, the paper's Figure 1 lacks clarity due to the absence of units on the y-axis. To enhance the readability and usefulness of this figure, it is advisable to not only include units but also consider revising the representation of time. Instead of using the week number of the year, incorporating reference dates that can easily pinpoint the months covered would significantly improve the ability to identify potential seasonal patterns within the data. Such a modification would make the figure more intuitive and facilitate a deeper understanding of the temporal dynamics being illustrated**

Response I have added the figure which I included dates with the month. I have added the in the figure 1 according to the reviewer's comments. it is stated in line 23, Introduction Section

In Fig 1 states the monthly change with specific dates can easily be visible where drifts are available.

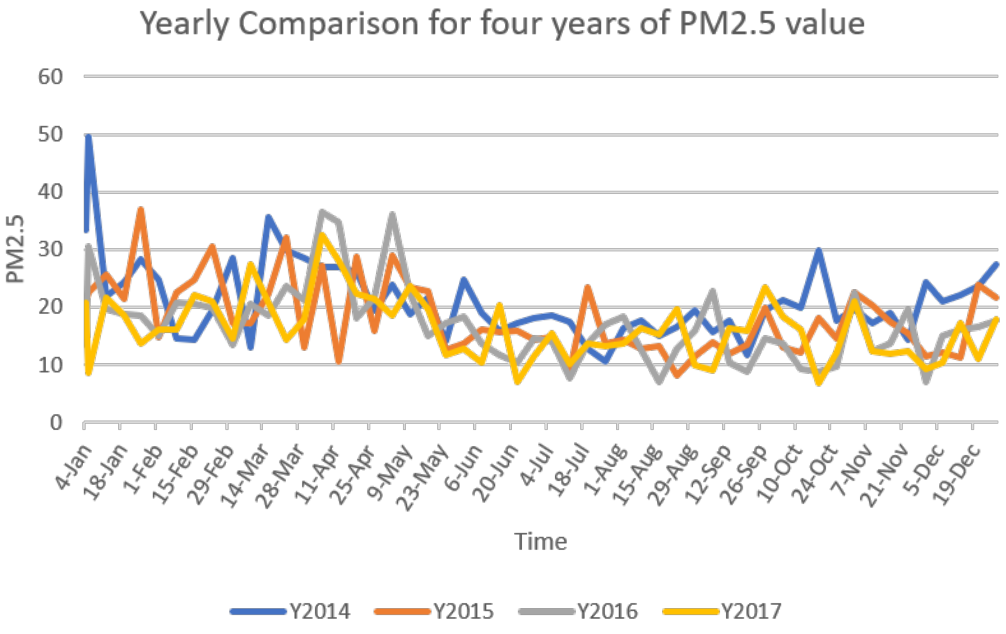

Fig 1. Weekly Average PM2.5 Levels of Years from 2014-2017

Comment **5) Specific Comment 5: Regarding Figure 2, which presents a comparison of the time series distributions, there is a notable absence of quantitative insights into the specific differences observed between the distributions. While visual comparisons can be informative, they leave much to the reader's interpretation and assumptions about the nature and significance of the differences. To address this, the paper should include a detailed analysis of the distributions, highlighting significant quantitative differences.**

Response I have explained the data distribution difference from the figure 2. It is discussed in line 35-39

~~Fig 2 shows the probability distributions of PM2.5 levels over various years, highlighting the noticeable differences between them. As the annual phenomenon of PM2.5 levels is evident, our study adopts an annual perspective as a basis to observe and investigate the prediction of PM2.5 levels.~~ Fig 2 shows the normal distributions of PM2.5 levels over various years, highlighting the noticeable differences between them. In Figure 2 it is shown that the yearly histogram can also check the

yearly comparison since the annual phenomenon of PM2.5 levels is evident, our study adopts an annual perspective as a basis for observation and investigation of the prediction of PM2.5 levels.

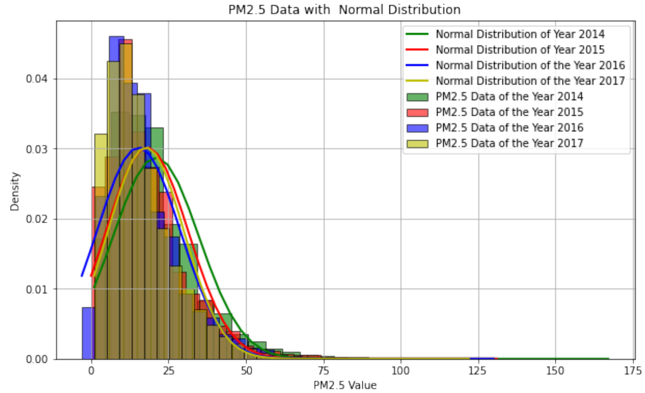

Fig 2. PM2.5 distributions from Year 2014 to 2017

Comment **6) Specific Comment 6: For Figures 3 to 6, where the results are presented, it's critical to address the issue of readability and interpretation posed by the extensive use of grouped bars. The dense clustering of bars makes it challenging to discern patterns or draw meaningful insights from the data. The authors are advised to consider alternative visualization techniques that can facilitate a clearer understanding of the results. One such recommendation is the use of heat maps to represent the average values, and/or the standard deviations of the different metrics found across all measurement points. Heat maps could provide a visually intuitive means to identify variations and patterns in the data, highlighting areas of interest more effectively than the current bar groupings. Another suggestion is to employ a matrix format that includes references to the years and represents the distribution shapes of the metrics found (e.g., probability functions or boxplots). This approach would allow for a more nuanced comparison across different years combinations, making it easier to identify where metrics are generally higher or lower. Furthermore, it's essential that all figures include titles on their axes.**

Response After getting the feedback from the reviewers, I have added the heat maps for each methodology and it has been shown from Fig 3-4 to Fig 4-5 and Fig 5-6 . In-Line included numbers are 253-254,267-268,277-278.

I have added the Heat map figure so changes can be easily identified from the different statistical tech-

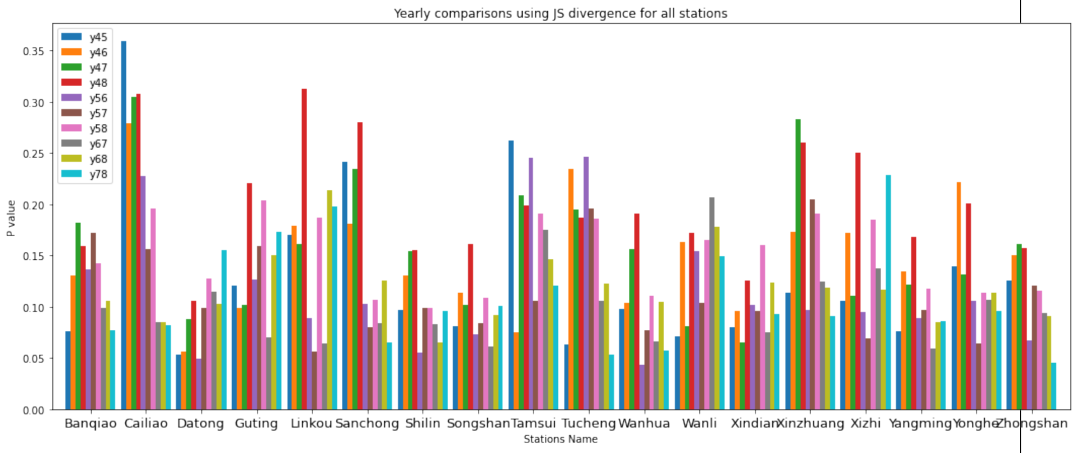

niques

Fig 3. Yearly comparisons using JS divergence for all stations

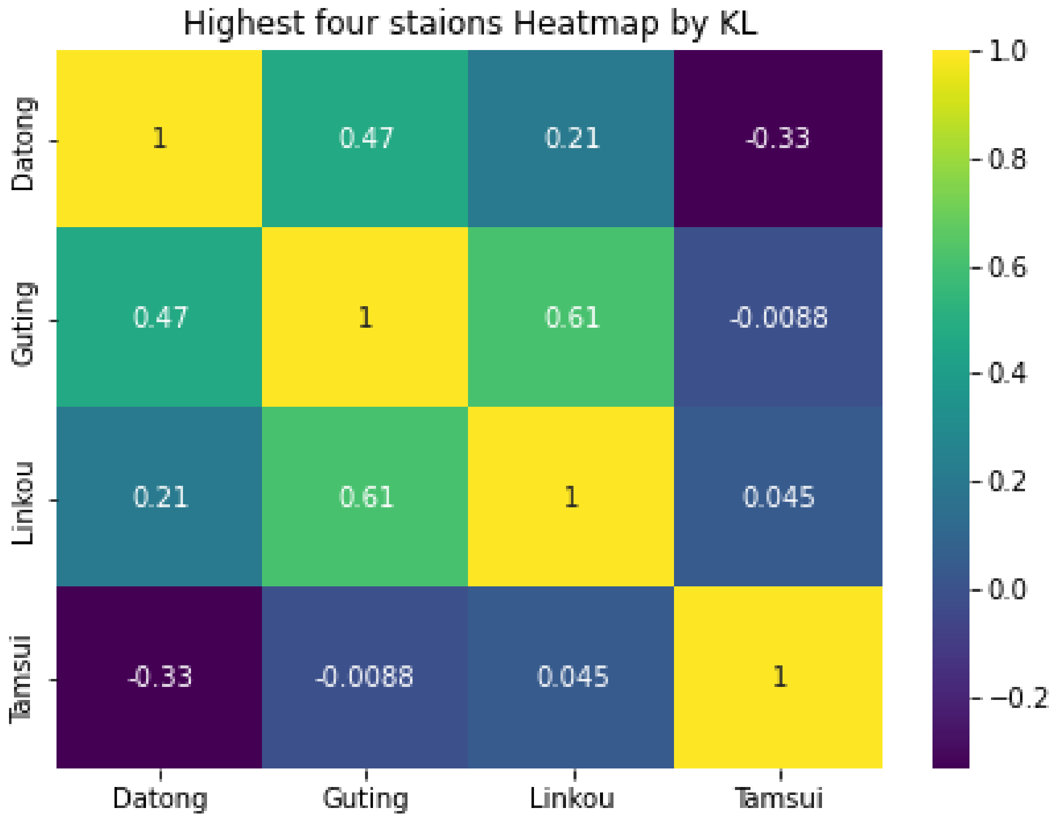

Fig 4. Yearly comparisons using JS divergence for four stations by heatmap

In Fig 4 includes the highest four stations P value by JS divergence, it is presented by heatmap diagram so can clearly understand the value differences, this value is calculated in the time of yearly comparison, here in Datong and Guting has maximum values.

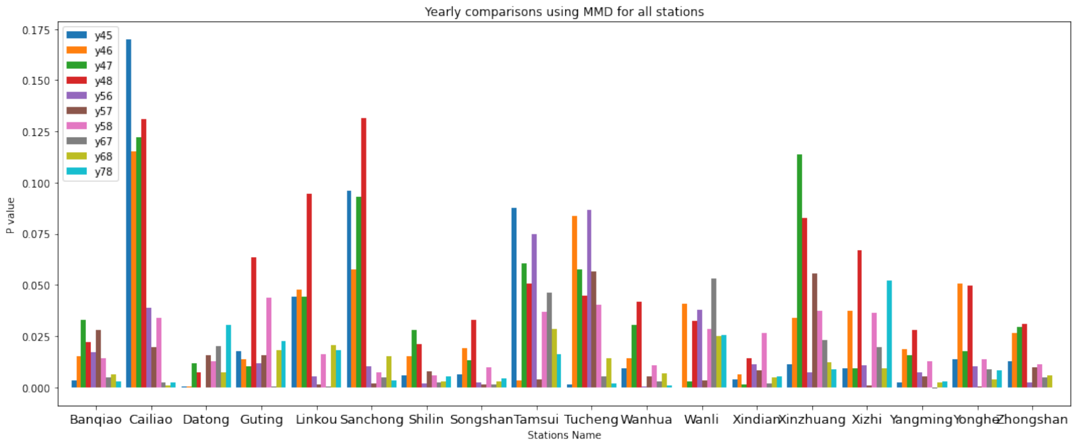

Fig 5. Yearly comparisons using Pearson Correaltion

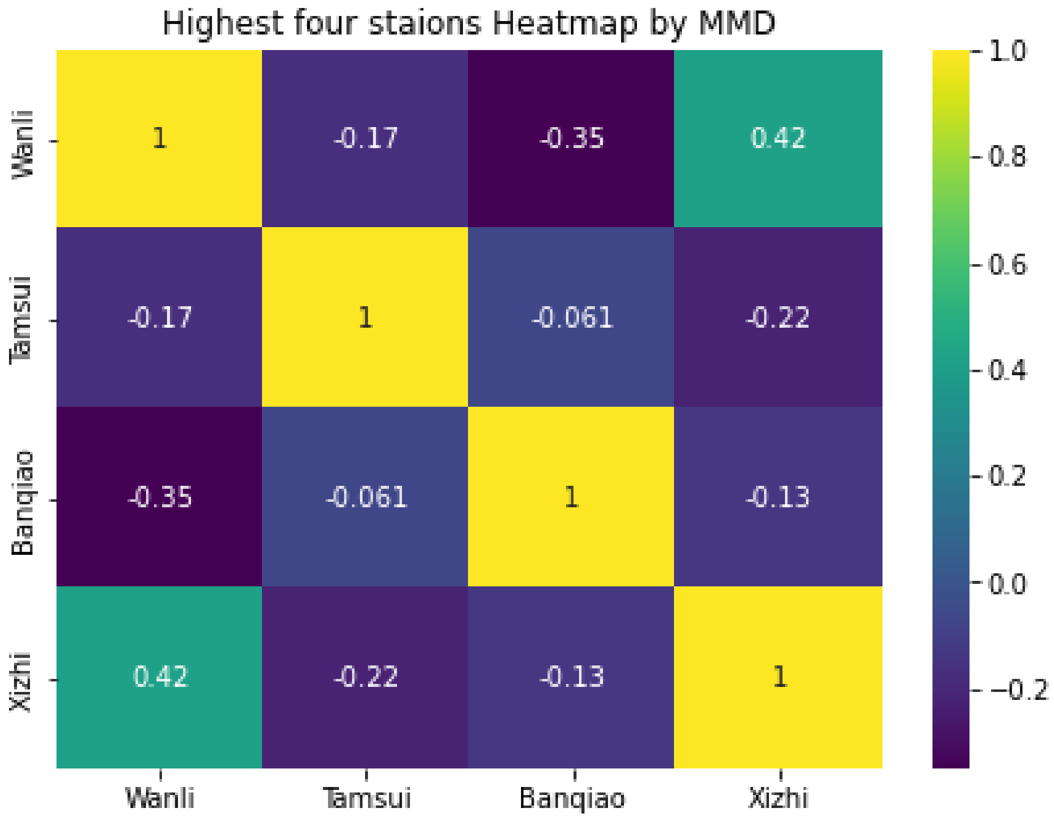

Fig 6. Yearly comparisons using PC for four stations by heatmap

In Fig 6 included the highest four stations of P value by MMD, it is presented by heatmap diagram so can clearly understand the value differences, here Wanli and Xizhi stations has maximum P value.

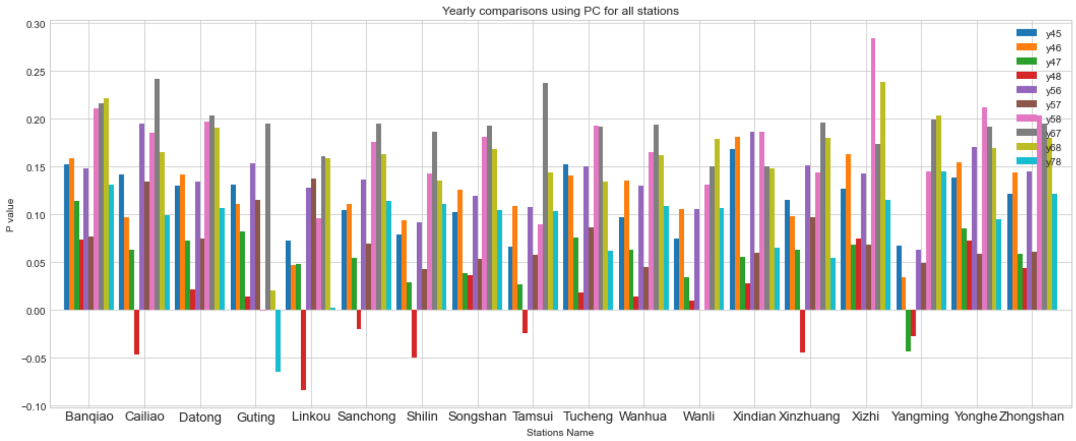

Fig 7. Yearly comparisons using MMD for all stations

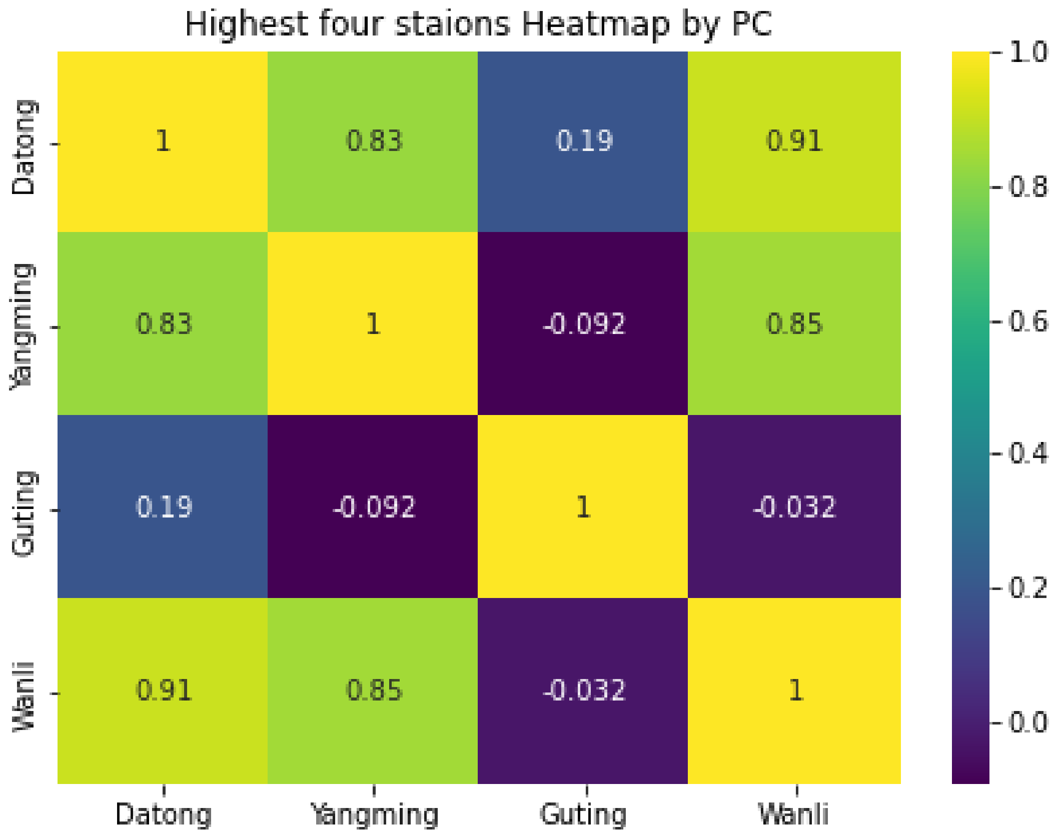

Fig 8. Yearly comparisons using MMD for four stations by heatmap **In Fig ?? included the highest four stations P value by PC, it is presented by heatmap diagram so can clearly understand the value differences, Datong and Wanli has maximum values.**

Comment **7) Specific Comment 7: For the presentation of time series forecasting results, it's indeed beneficial to go beyond the use of Root Mean Square Error (RMSE) as the sole metric of accuracy. Including comparisons using Mean Absolute Error (MAE) and Mean Absolute Percentage Error (MAPE) is recommended, as these metrics are also widely utilized in the field and provide different perspectives on forecast accuracy.**

Response Thank you for your advice. After getting the advice of the reviewers, I have added the RMSE, MAE, and MAPE results for PM2.5 predictions. In the Evalution Criterian section I discussed its definition. And the Results and Discussion section I discussed about its MAE and MAPE results.

To evaluate the performance of the baseline models and the proposed model, we have applied different methods such as the root mean square error (RMSE), mean absolute error (MAE), and mean absolute percentage error (MAPE) to evaluate their performance. We have shown their results in Results Section.

Comment **8) Specific Comment 8: Lastly, it's paramount to ensure clarity and consistency in the notation used throughout the paper, especially in the mathematical equations presented. Every symbol, variable, and function within these equations should be explicitly defined and described within the text.**

Response I have carefully checked the consistency of the notation used in this paper, and also the equations.

4. Reviewer 2

Comment **Reviewer 2: (general comment) 1. The last sentence of the abstract is not complete.**

Response There was a mistake. I have completed the first line.

> Additionally, we introduced a wrapped loss function incorporated into a model, resulting in more accurate results compared to those using the original loss function alone..

Comment **Reviewer 2: (general comment) For the readers' advantage, they need to elaborate on the point description of the proposed FLC and BLC models along with better and more comprehensive/professional-looking diagrams. In its present form, the paper looks like copy pasted from a scientific report or a thesis.**

Response After getting feedback from the reviewer, I have redrawn it following the advice. It is redrawn in the section in Model Designs in the subsection in FLC and BLC models.

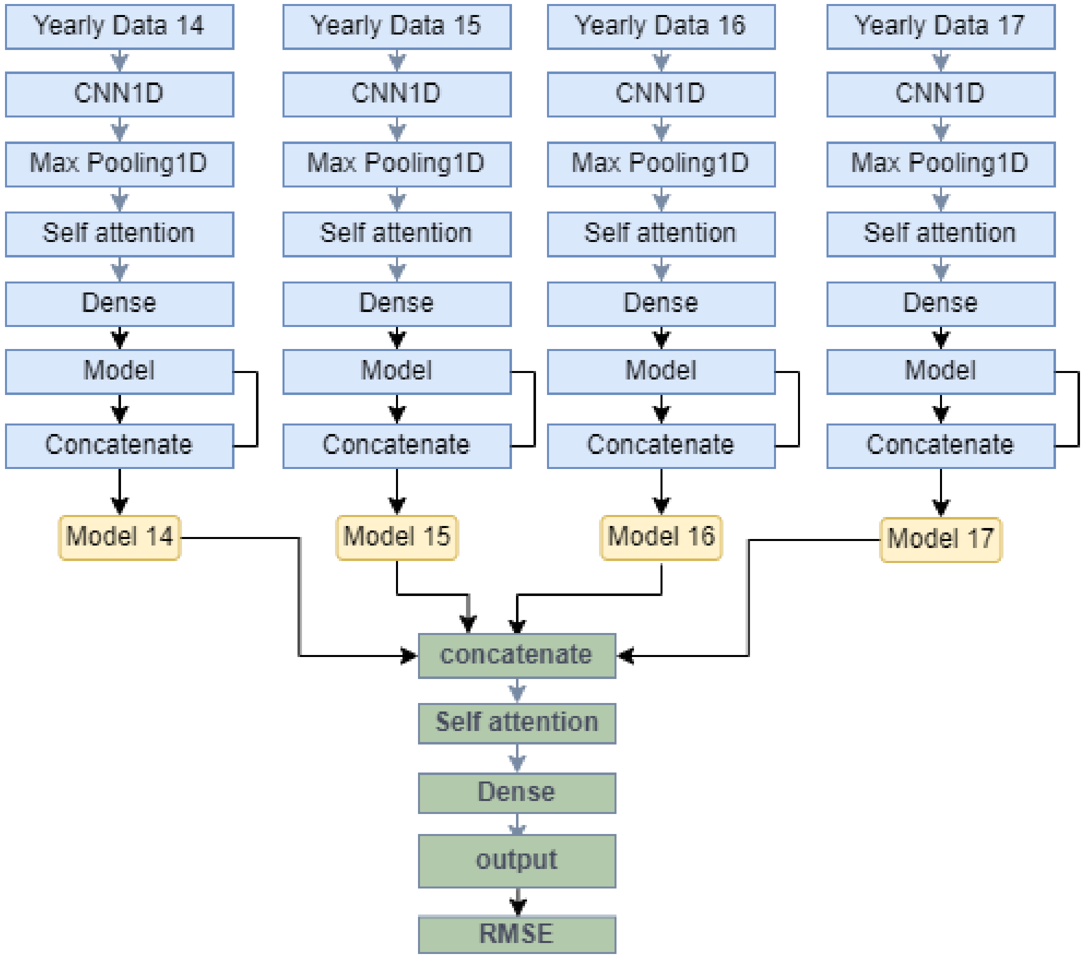

Fig 10. Front-loaded connection model

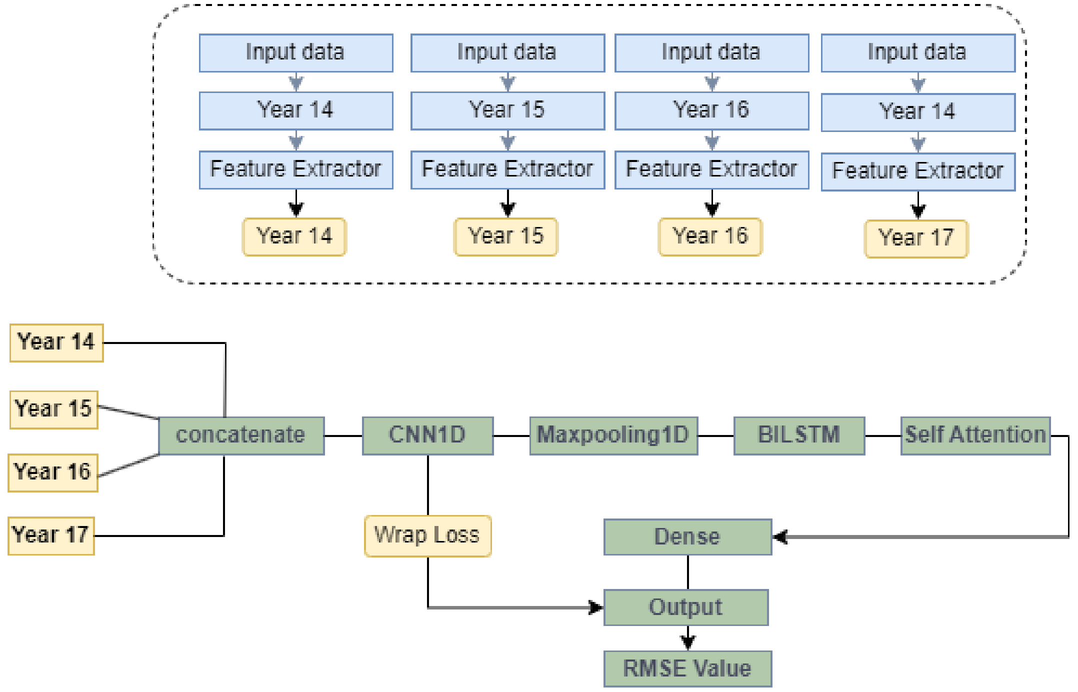

Fig 11. Back-loaded connection model

Comment **Specific Comment 2-1: In the title they mention the use of Deep NN, but in the model description they mention CNN. Why they have not mentioned CNN in the title?**

Response I have edited my title according to the feedback from reviewers.

> On the Data Shifting: Predicting PM2.5 levels by using Enhanced Deep neural network ~~enhanced based nural network~~On the Data Shifting: Predicting PM2.5 levels using CNN-based neural network

Comment **Specific Comment 2-2: In the Model Design Section (FLC and BLC), they mentioned the conclusion along with a description (although inadequate) of the models (in lines 320-322: The results clearly demonstrate that both the front-loaded and back-loaded models proposed outperform other models, and the wrapped model exhibits superior performance compared to the one without the wrapped loss function). Is it required here?**

Response I have replaced the sentence that was there in lines 320-322.

> ~~The results clearly demonstrate that both the front-loaded and back-loaded models proposed outperform other models, and the wrapped model exhibits superior performance compared to the one without the wrapped loss function~~The results clearly show that Back loaded models perform well compared to other models and warp loss when added to the model after that assists in increasing the model performances too that is also included on figure 12

Comment **Specific Comment 3: A complete description of the datasets created/used should be given in its section before the Experimental setup.**

Response I have added the details description of our datasets for evaluation in lines 332-339

In this work, the 2014-2017 yearly data are used for model training and the 2018 yearly data for model testing. The air quality datasets were collected from the Taiwan EPA website (data.epa.gov.tw). The EPA website has PM2.5, PM10, carbon monoxide (CO), nitric oxide (NO), nitrogen dioxide (NO2), nitrogen oxides (NOx), ozone (O3) and sulfur dioxide(SO2). The hourly meteorological data were collected from the Weather Bureau website (CWB) (opendata.cwb.gov.tw). The dataset, include humidity, temperature, pressure, rainfall, and wind speed and direction.

Comment **Specific Comment 4: The results of the Experiments should be illustrated in a separate Results Section.**

Response Thank you for your feedback. I have added the Result section in my work where I have discussed the result.

The results clearly demonstrate that both the front-loaded and back-loaded models proposed outperform other models, and the wrapped model exhibits superior performance compared to the one without the wrapped loss function .

Comment **Specific Comment 5: Results should be discussed appropriately in a separate Discussion Section.**

Response I have added a new section called Results and Discussion. The results for MAE and MAPE are discussed in the discussion section.

Discussion for MAE value From Fig ,it is revealed that the proposed model BLC with Wrap loss performs well compared to all other models like WBILSTM is better compared to BILSTM. It is shown that there are LSTM, BILSTM, CNN models performing well but not well compared to our proposed model BLC models. We have applied LSTM attention, but it is not performing well compared to our proposed BLC model. The detailed results for the Sanchong station are given in Table where it is observed that the MAE values increase when the number of hours increases. Compared with all models, it is clearly shown that the BLC with Wrap loss and FLC with wrap loss are performing well. From Fig 15, it is shown that the MAPE percentage for our proposed models is quite low compared to other models. Compared with all other models, the wrap loss with BLC has a minimum MAPE for 64h prediction. In Table 4, it shows that the MAPE percentages increase accordingly after increasing the number of hours. In Table 5, it includes the models BILSTM, WBILSTM, LSTM , WLSTM, LSTM-AT, WLSTM-AT, CNN, and WCNN models. FLC and BLC performed well compared to other models. Among all models, it is shown that the BLC with the wrap model has less MAPE percentage. Here, a lower percentage value means that it works well.

Comment **Specific Comment 6: The Conclusions should be drawn as per the findings in the research work and they should be substantiated with the outcomes.**

Response I have concluded with the outcomes of the work described in the Conclusions section.

In this work, it is calculated the data drifting scenario is calculated from the data of the last 5 years using different statistical techniques. After getting the P-value from those techniques, the highest and lowest data drifting stations are mentioned from the datasets. We have applied the traditional model for predicting the PM2.5 hourly prediction and we have applied the LSTM, BILSTM and CNN models for hourly predictions. There is a contribution to propose the wrap loss which assists the model in increasing the model performances, and it is applied to all the baseline models and models with wrap loss got better results compared with baseline models. After considering the previous year's datasets and transferring the knowledge to next year's predictions, we proposed the FLC and BLC models. Among them, the BLC model with wrap loss has achieved good results as demonstrated by calculating

bt RMSE, MAE, and MAPE values.

Comment **Specific Comment 7: The organization to present the paper needs to be improved. They may read the papers to improve the presentation. https://doi.org/10.3390/diagnostics12051134 https://ieeexplore.ieee.org/stamp/stamp.jsp?arnu https://ieeexplore.ieee.org/stamp/stamp.jsp?arnumber=9956807 At the end of the Introduction section, describe the organization of the paper in brief.**

Response I have read those papers and changed my introduction section by including the organization of the paper according to upper mentioned papers.In Lines 65-74 and also improved the contribution in lines 85-89 and 93-96.

It is mandatory to know the pollution data scenario and which location data has drifted, most recently the research work about the data drifting is very limited, so knowing about the datasets will help to take prerequisite steps. To increase the model performance, many researchers proposed the weighted loss function to be added with the model training process, the loss function can deal with increased model performances. The loss function can be a solution for a data drifting scenario. It is also needed to find a model that will consider the last couple of years' data to predict the hourly PM2.5 predictions. As we can collect the last couple of years' data, we can design a model that can transfer their knowledge to predict the next year's hourly predictions. The proposed model handles annual data separately and employs the wrapped loss function to learn the difference between different data for the year. However, we have evaluated several baseline models such as BILSTM, LSTM, CNN, and LSTM-attention to evaluate their performance for hourly PM2.5 predictions. An effective loss function has been proposed that can improve model performance in data training time in any model. The evaluation of PM2.5 hourly prediction performance was carried out by calculating the Root Mean Square Error (RMSE), and Mean Absolute Error(MAE), Mean Absolute Percentage Error(MAPE).

Comment **Specific Comment 8: The purpose of using many statistical methods should be properly explained in the paper.**

Response I have discussed the statistical methods explained in previous work.

Comment **Specific Comment 9: A comparison of the models with SOTA-published works should be given.**

Response I have included the model applied with references in the work. Details in "Model designs" sections and compare their results in the Result sections.

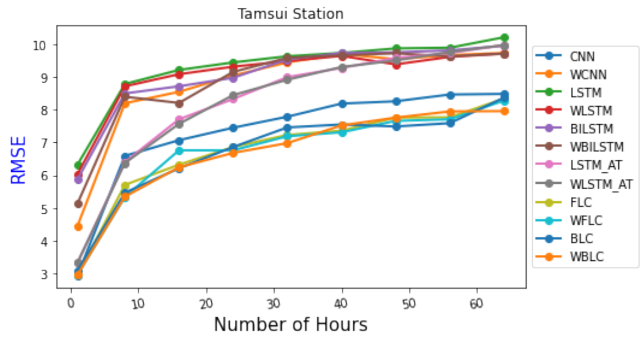

Fig 12. RMSE of different models for Tamsui

Comment **Specific Comment 10: The related Work/literature review section should be more elaborate to show the substantial research gaps and accordingly the research problem statement should be mentioned after the Introduction section.**

Response I have added there the substantial research problem and research gap after the Introduction Section. I have added in Previous work sections in lines 104-110,117-122,157-160.

> In this work [22], they focused on learning the relation between PM2.5 concentrations and climate variables and also considered future context data sequences for model training, also encountering the correlation between air pollutants and climate variables. They did not work on yearly data drifting or changing scenarios; moreover, they focused on future context but did not consider the previous year's data. As RNN-based networks did not consider how the model loss function can work efficiently to solve these issues, we propose a model with a loss function and also show the way to calculate data drifting in the datasets. The authors [28] used target data and nearby monitoring station data to predict PM2.5 data. They did not consider the drifting scenario of the data, and also they did not work with the previous year's data for yearly prediction. The author did not work with the model training process on how to increase the model performance by proposing the loss function, they focused on seasonal variability and nearby stations and also summarized the wind directions. The authors proposed a new novel and multi-task that can easily learn the various classification and regression losses by solving homoscedastic tasks and designing a semantic segmentation architecture, but did not consider how it can work more perfectly in a single optimal weighting for all tasks. They did not propose a unified model for PM2.5 hourly prediction to consider with data drifting which can produce more good results for time series data.
