# Supplementary material for: Enhancing PM2.5 prediction by mitigating annual data drift using wrapped loss and neural networks
Source: PLoS One. 2025 Feb 11;20(2):e0314327. doi: 10.1371/journal.pone.0314327 (PMC11813127; doi:10.1371/journal.pone.0314327)
Supplement: Attachment — Submitted filename: Response to Reviewers.pdf [file pone.0314327.s004.pdf]

[Responses Academic Editors and Reviewers]

Point-by-Point Responses and the Reviewer Comments of

On the Data Shifting: Predicting PM2.5 levels by using CNN based neural network

Md Khalid Hossen*, Yan-Tsung Peng, Meng Chang Chen

Plos Reviewers Response

Comment **1: Abstract is not clear about what the paper proposes.**

Response After getting the feedback,I have written it, and elaborated on the purposes of my work.

Comment **2. The paper needs to clearly outline the main contributions are, such as analysis of data drifting, drift detection, deep learning model (FLC and BLC Models), or the application of wrapped loss function in the models.**

Response I have written the main contributions of the paper in the introduction section

Comment **3. The introduction should clearly and explicitly describe the problem statement and the contributions.**

Response I have rewritten the problem statement and contribution clearly in the Introduction section.

Comment **4. In Previous work section, the paper frequently uses the subject "authors". It is unclear whether this refers to the authors of the current paper or the authors cited in the references.**

Response I have edited the authors from the paper and stated clearly with the author's name of the paper.

Comment **5. In lines 112-119 in page 4, the statement "Among them, CNN-based models stand out for their 115 ability to process historical data from EPA stations based on the spatial distribution of 116 the sites [28]" attempts to conclude that CNN is better based on [28]. Among various models for various data in refs [22-28], it would be better to clearly explain why CNN is preferred, supported by strong evidence.**

Response After getting feedback, I have included a few works where that applied CNN based model for PM2.5 predictions the lines are given below from 119-124.

Comment **6.The Previous work section does not discuss the attention-base model which is proposed by the paper. .**

Response I have discussed the attention base model on the previous work section.

Comment **7. From Fig 3 to Fig 8, it is unclear what the y-axis values represent. Are they the results from Eq (3), (4), (5), and (6) or just P-value? It is important to note that the interpretations of the results from PC differ from those of other methods. From Fig 3 to Fig 8 the results are from equations 3-6, those equations calculated the P value finally. The interpretation of those results is given there.**

Response From the equation (3) Eq (3), (4), (5), and (6), calculated the P value and value are illustrated on Figure 3-8. Heatmaps are based on the P value that we get after those equations are calculated.

Comment **8. In page 12, it is better to explain FLC and BLC models in more detail.**

Response The model description, I have described those models in more detail in FLC and BLC models sections.

Comment **9. It is strongly recommended to reorganize the paper.**

Response I organize the paper according to feedback.

1. Plos second Reviewers Response

Comment **o Line 98-203: I think there is no need of this Section giving too much theoretical information. It could be more appropriate for a review paper. It is better to shorten this part and include as part of the introduction.**

Response I have edited the section and shortened it as I needed to discuss the previous work so I kept it and the another reviewer also asked to introduce the applied model in details..

Comment **o Add a section on Study Area and Data. Give details about the study area with Map with location of various monitoring stations.**

Response I have added a section in the name Study area and data and drawn a map on the location and described it.

Comment **o Include a Section on Methods giving full details in sequence about the method used in the study. A typical machine learning related work should include section as Data collection and pre-processing, EDA and Feature engineering, Model development and evaluation, and finally prediction.**

Response I have discussed the data collection and analysis of how collected the data in Data collection and pre-processing section . There are several section as Model development and prediction .

Comment **o . In figures, the default design for axes, labels, and legend has been used. Make label bold and enlarge.**

Response I have redrawn the figures and made them bold too accordingly.

Comment **o Write which software packages or libraries were used for the statistical analysis. Give references too.**

Response I have given the references for the work's statistical analysis as there are three techniques so I gave all of them references too.

Comment **o In conclusion, add a few sentences on policy implications and future work. Add limitations here.**

Response I have added future work and limitations too in the following paragraph.
