# Supplementary material for: Enhancing PM2.5 prediction by mitigating annual data drift using wrapped loss and neural networks
Source: PLoS One. 2025 Feb 11;20(2):e0314327. doi: 10.1371/journal.pone.0314327 (PMC11813127; doi:10.1371/journal.pone.0314327)
Supplement: Attachment — Submitted filename: Response to Reviewers.pdf [file pone.0314327.s005.pdf]

[Responses Academic Editors and Reviewers]

Point-by-Point Responses and the Reviewer Comments of

Enhancing PM2.5 Prediction by Mitigating Annual Data Drift Using Wrapped Loss and Neural Networks

Md Khalid Hossen*, Yan-Tsung Peng, Meng Chang Chen

Plos Reviewers Response

Comment **1: Please ensure your figures have sufficient description. The description for most figures is too short. You will also want to provide units for PM2.5, which I could not locate in figure captions or in many parts of the text. (i.e. MAE is in the units of PM2.5 concentration, but units are not listed)**

Response After getting the feedback, I have included the units for MAE $(\mu g/m^3)$, PM2.5$(\mu g/m^3)$ and unit for RMSE $(\mu g/m^3)$ too. I have included in the text and in their figure too.

Comment **2. consider a revision to your manuscript title. As it is, the title does not inform the reader about all of the major work that you've completed and is not representative of the manuscript. Current title: "On the Data Shifting: Predicting PM2.5 levels by using CNN based neural network". My critique is that "data shifting" is too vague and you've done more than just use CNNs. You should revise the title, a potential recommendation could be something like "Improving PM2.5 pollution predictions by correcting for annual data drift with wrapped loss functions evaluated against CNNs and LSTMs". Please feel free to use the suggested or modify as you like.**

Response Thank you for your suggestion and I modify a little .The current tile are given below "Enhancing PM2.5 Prediction by Mitigating Annual Data Drift Using Wrapped Loss and Neural Networks".

Comment **3. Please check over the text for readability and English in the manuscript. For example, the use of "yanking" in the abstract is confusing and that sentence should be reworked.**

Response I have revoked the sentence. I have checked the text and readibilty and modified accordingly.

Comment **4. Consider adding specific major metrics in your conclusion and abstract sections (i.e. how much did your wrapped loss function improve on the accuracy when dealing with data drift?); this will increase audience interest and usability of your findings.**

Response Thank you for your suggestions. I have included the model performance percentage how much they improved it and included in the Abstract and conclusion.

Comment **5. Ensure abbreviations and terminology are consistent throughout the manuscript (i.e. p-value vs P value).**

Response After getting feedback, I have fixed the manuscript only P value .

Comment **6. Lastly, keywords. Considering adding more if allowed. Additionally, consider related terms beyond abbreviations.**

Response I have added more keyword .
