# Supplementary material for: Enhancing PM2.5 prediction by mitigating annual data drift using wrapped loss and neural networks
Source: PLoS One. 2025 Feb 11;20(2):e0314327. doi: 10.1371/journal.pone.0314327 (PMC11813127; doi:10.1371/journal.pone.0314327)
Supplement: Attachment — Submitted filename: reviewer_comments.docx [file pone.0314327.s003.docx]

**Reviewer’s Recommendation and Comments for**

**Manuscript ID PONE-D-24-05738**

**Title**: “On the Data Shifting: Predicting PM2.5 levels by using Enhanced Deep neural network”

**Recommendation:**

I think that the manuscript can be considered for publication after minor revisions.

Specific comments are given below:

**Comments**

- Line 98-203: I think there is no need of this Section giving too much theoretical information. It could be more appropriate for a review paper. It is better to shorten this part and include as part of the introduction.
- Add a section on Study Area and Data. Give details about the study area with Map with location of various monitoring stations.
- Include a Section on Methods giving full details in sequence about the method used in the study. A typical machine learning related work should include section as Data collection and pre-processing, EDA and Feature engineering, Model development and evaluation, and finally prediction.
- . In figures, the default design for axes, labels, and legend has been used. Make label bold and enlarge
- Write which software packages or libraries were used for the statistical analysis. Give references too.
- In conclusion, add a few sentences on policy implications and future work. Add limitations here.
